# Exploring the sustainable elimination of dye using cellulose nanofibrils- vinyl resin based nanofiltration membranes

**DOI:** 10.1186/s13065-024-01211-5

**Published:** 2024-06-27

**Authors:** Ahmed H. Ragab, Najla F. Gumaah, Aya Abd El Aziz Elfiky, Mahmoud F. Mubarak

**Affiliations:** 1 Chemistry Department, College of Science, King Khalid University, 61413 Abha, Saudi Arabia; 2 Chemistry Department, Faculty of Science, Northern border university, Arar, Saudi Arabia; 3 Petrolum Applications Department, Egyptian Petroleum Research Institute (EPRI), Ahmed El-Zomer, Nasr City, Cairo Egypt; 4 Core lab center, Egyptian petroleum research institute (EPRI), 1 Ahmed El Zomor St, Nasr City, 11727 Cairo Egypt

**Keywords:** Self-cleaning, Nanofiltration, Methylene blue, Industrial wastewater, Adsorption, Sustainable

## Abstract

This study focuses on the development of a novel self-cleaning nanofiltration membrane for the efficient removal of the cationic dye methylene blue (MB) from industrial wastewater. The membrane is composed of vinyl resin (VR), cellulose nanofibrils (CNF), and titanium alpha aluminate (TAAL) nanoparticles.

The TAAL loading ranged from 1 to 5 wt%, the pH varied from 5 to 10, and the initial MB concentration ranged from 10 to 50 ppm. Using a dead-end filtration system, the (VR/CNF@TAAL) membrane with 5 wt% TAAL at pH 10 demonstrated excellent performances. It achieved a remarkable 98.6% removal efficiency for 30 ppm MB dye, with a maximum adsorption capacity of 125.8 mg/g. The adsorption kinetics analysis revealed that the process followed the pseudo-second-order model, indicating a chemisorption mechanism. The rate constant was determined to be 1.2732 × 10^–3^ g mg^−1^ min^−1^. The Freundlich isotherm model provided a better fit (R^2^ = 0.996) than the Langmuir model, suggesting multilayer adsorption on the nanocomposite membrane surface. In addition to its high adsorption and filtration capabilities, the (VR/CNF@TAAL) nanocomposite membrane exhibited cost-effectiveness and environmental friendliness as an adsorbent for MB removal from industrial wastewater. The membrane’s self-cleaning property further contributes to sustainability by reducing the need for additional chemical treatments.

## Introduction

The discharge of wastewater containing cationic dyes has become a significant environmental concern, leading to the development of sustainable and efficient methods for their removal [31]. The result of nanofiltration membranes has gained considerable attention in recent years, offering a promising solution for the removal of pollutants from wastewater. In this study, we present a novel self-cleaning nanofiltration membrane made of microcrystalline nanocellulose and titanium aluminate nanoparticles embedded in vinyl resin (VR) for the removal of cationic dyes from wastewater [5].

Dye removal from wastewater is an important environmental challenge due to the toxicity and visibility of dye molecules. Conventional treatment methods such as coagulation, precipitation, adsorption and membrane filtration have been extensively studied for dye removal.

Adsorption using low-cost adsorbents is an attractive option due to its simplicity and high efficiency. Various agricultural and industrial wastes have been explored as adsorbents including chitosan, peat moss, bentonite and activated carbon. However, adsorbents need to be regenerated or disposed after saturation, increasing processing costs.

Membrane filtration offers an alternative through continuous separation without regeneration. Nanocomposite membranes integrating nanomaterials into a polymer matrix have received attention due to their enhanced selectivity and antifouling properties compared to pure polymer membranes. Metal oxides such as titanium dioxide, aluminum oxide and zinc oxide are commonly used fillers to impart hydrophilicity and charged surfaces for dye adsorption.

While significant progress has been made, developing cost-effective and reusable membrane materials remains an active area of research. In this work, we build upon previous studies by synthesizing a novel PVC/microcrystalline nanocellulose@titanium aluminate membrane integrating adsorption capabilities with self-cleaning function for sustainable dye removal. The unique membrane characteristics and fouling resistance are systematically investigated.

Conventional wastewater treatment processes, such as coagulation, sedimentation, and biological treatment, are widely employed for the removal of various pollutants, including dyes. However, these processes often face limitations in effectively removing recalcitrant and persistent dyes, particularly cationic dyes like methylene blue (MB). Furthermore, these methods can generate large volumes of sludge, require extensive treatment facilities, and involve complex operations, leading to high costs and potential environmental concerns.

The novelty of our work lies in the incorporation of cellulose nano fibrils and titanium alpha aluminate nanoparticles into VR to develop a membrane with enhanced adsorption and filtration capabilities. The synthesized membrane was designed with a unique self-cleaning property that allows for the easy removal of fouling agents, thereby maintaining long-term stability and high filtration performance. We investigate the membrane’s adsorption efficiency towards cationic dye MB under various conditions, including adsorbent dosage, pH values, and dye concentrations [26].

The main contribution of our study is the successful development of a self-cleaning PVC/cellulose nanofibrils (CNF)@titanium alpha aluminate nanofiltration membrane with high efficiency in removing cationic dyes from wastewater. The membrane’s unique self-cleaning property allows for the easy removal of fouling agents, maintaining long-term stability and high filtration performance. Additionally, we explore the kinetics and isotherm models of the adsorption process, providing insights into the behavior of the membrane and its potential applications in wastewater treatment [13].

From an industrial application point of view, the self-cleaning VR/cellulose nanofibrils @titanium alpha aluminate nanofiltration membrane has significant potential for the efficient removal of cationic dyes from wastewater in various industrial settings, such as textile, printing, and dyeing industries. The membrane’s high efficiency, stability, and reusability make it a promising candidate for large-scale industrial applications, contributing to the sustainable development of our society [34]. The use of this novel membrane material can significantly reduce the operational costs and environmental impact of dye removal from wastewater [10].

The practical consequences of our work include the potential for industrial applications and significant environmental benefits, contributing to the sustainable development of our society. The use of this novel membrane material can significantly reduce the operational costs and environmental impact of dye removal from wastewater. Moreover, the findings of this study provide valuable insights for other scientists working in the field of wastewater treatment and nanofiltration membrane development [20].

This work presents a novel approach to addressing the efficient removal of cationic dyes from wastewater through the development of a self-cleaning nanofiltration membrane. The membrane is composed of vinyl resin (VR), cellulose nanofibrils (CNF), and titanium Alpha aluminate (TAAL) nanoparticles. By incorporating CNF and TAAL into the VR matrix, a nanocomposite membrane is created with enhanced adsorption and filtration capabilities compared to conventional VR membranes.

The use of environmentally friendly and cost-effective CNF and TAAL allows the nanocomposite membrane to achieve high adsorption capacity and removal efficiency for cationic dyes, such as methylene blue. The membrane’s self-cleaning property is facilitated by the hydrophilic nature of CNF and TAAL, enabling easy removal of fouling agents and ensuring long-term filtration performance.

Extensive investigations have been conducted to understand the membrane's adsorption behavior. Factors such as TAAL loading, pH, and initial dye concentration were studied to determine their effects on adsorption capacity and removal efficiency. The results indicate that the membrane follows the pseudo-second-order model for adsorption kinetics, suggesting a chemisorption mechanism. Additionally, the Freundlich isotherm model describes multilayer adsorption on the nanocomposite membrane surface.

Furthermore, the membrane’s potential for industrial applications in textile, printing, and dyeing industries has been demonstrated. It offers a sustainable solution for wastewater treatment and dye removal, contributing to environmental preservation and addressing economic concerns. The unique composition, high performance, and reusability of the self-cleaning VR-CNF@TAAL nanocomposite membrane make it a promising option for efficient cationic dye removal from wastewater.

This paper aims to present a novel approach to developing a self-cleaning nanofiltration membrane using microcrystalline nanocellulose and titanium aluminate nanoparticles embedded in VR for the removal of cationic dyes from wastewater. Our study provides a comprehensive investigation of the membrane’s adsorption ability towards cationic dye MB under various conditions. The successful development of a self-cleaning VR/cellulose nanofibrils@titanium alpha aluminate nanofiltration membrane with high efficiency in removing cationic dyes from wastewater represents a significant advancement in the field of wastewater treatment. The membrane’s unique self-cleaning property, high efficiency, stability, and reusability make it a promising candidate for large-scale industrial applications, contributing to the sustainable development of our society.

## Experimental

### Materials

Vinyl resin (VR), tetrahydrofuran (THF), dimethyl formamide (DMF), sodium hydroxide (NaOH), titanium acetate Ti (CH3COO)_2_. 2H_2_O, ammonium hydroxide NH_4_OH, titanium chloride (TiCl_4_), and methylene blue (MB) were received from ABC Chemicals. Distilled water used in this study was obtained from a local water purification system. Other chemical products utilized included distilled water, HCl, isopropyl alcohol, and NH_4_OH (25%) solutions acquired from ABC Chemicals. In this research project, all chemicals were of analytical grade. The agricultural wastes used as the microcrystalline cellulose source were obtained from farms in Egypt.

### Preparation of nanocellulose from purified agricultural wastes

- Stage 1: Unmasking the Raw Material:

The adventure begins with the Tetra Pak, shredded and stripped bare. A 24-h soak in distilled water softens its defenses, followed by a high-speed blending to unlock its fibrous secrets. The resulting suspension is filtered and washed, revealing the raw cellulose beneath, free of impurities and neutral in pH. Finally, gentle drying at 50 °C for a day unveils the raw cellulose, ready for its next transformation.

- Stage 2: Purification—Refining the Rough Diamond:

The raw cellulose, though liberated, still holds traces of unwanted elements. To refine it, a two-step NaOH treatment takes center stage. First, a 2% NaOH solution bathes the cellulose at 90 °C for 2 h, loosening and dissolving impurities. Neutralizing washes and filtering follow, leaving a cleaner version behind. This process repeats with a 5% NaOH solution, further polishing the cellulose until it shines.

- Stage 3: Bleaching—Lightening the Shadows:

But there’s more to purification than just removing impurities. A final bleaching step awaits, where the cellulose encounters a solution of acetic acid and sodium chlorite at 75 °C for 3 h. This powerful duo eliminates any lingering color, leaving the cellulose pristine and white, ready for its final transformation.

- Stage 4: Hydrolysis—Breaking Down to Build Up:

Now comes the moment of truth: hydrolysis. The purified cellulose faces a powerful challenger—64% sulfuric acid at 45 °C for 1–3 h. This potent acid breaks down the cellulose structure into tiny fragments, laying the foundation for the nanocrystals to emerge. Once the acid’s work is done, cold water dilutes the solution, and a neutralizing bath with NaOH restores balance. The resulting suspension then undergoes centrifugation, separating the precious cellulose nanocrystals from the remaining fragments. Finally, dialysis against distilled water for a week, followed by sonication and another round of centrifugation, purifies and isolates the nanocrystals, ready for characterization [29].

**Fig. 1:**
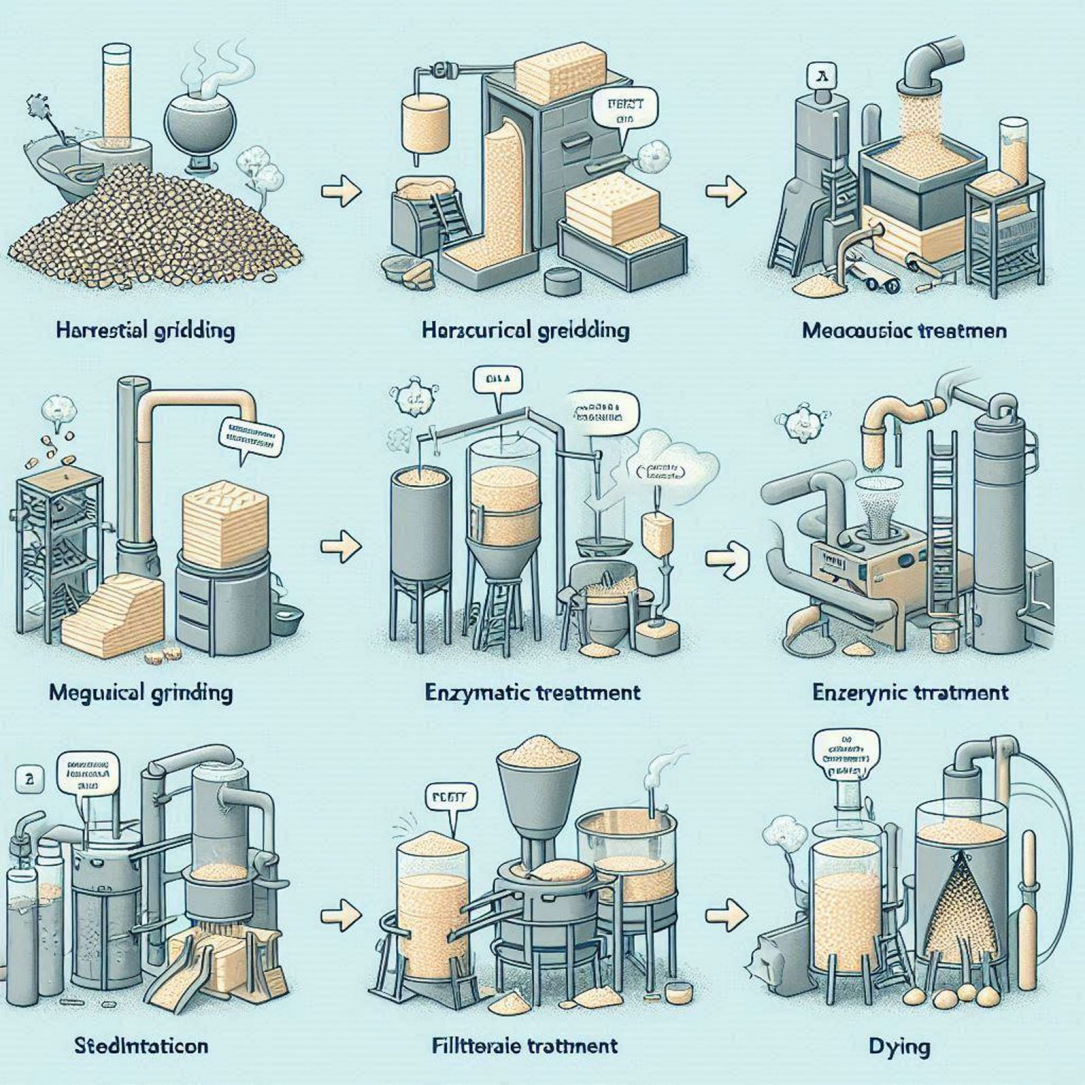
Synthesis of nanocellulose from purified agricultural wastes

- Stage 5: Characterization—Unveiling the Secrets:

The journey culminates in revealing the true nature of these microscopic wonders. Powerful techniques like XRD, FTIR, TEM, and TGA unveil their secrets. XRD analyzes their crystalline structure, FTIR explores their chemical composition, TEM provides a glimpse of their stunningly small size and shape, and TGA reveals their thermal stability as shown in Fig. 1 [33].

### Synthesis of metal oxide nanomaterials

The fabrication and characterization of cellulose-TiO_2_ composite film for desalination involves several materials and methods. The materials required for the process include cellulose nanofibrils (CNFs), titanium dioxide (TiO_2_) nanoparticles, deionized (DI) water, sodium alginate (SA), polyethylene glycol (PEG) as an optional additive, and calcium chloride (CaCl_2_).

The first step is the preparation of the cellulose-TiO_2_ composite. This involves dispersing 0.5 g of CNFs in 50 mL of DI water with magnetic stirring for 1 h. Similarly, 0.25 g of TiO_2_ nanoparticles are dispersed in 50 mL of DI water with magnetic stirring for 1 h. The TiO_2_ dispersion is then combined with the CNF dispersion and stirred for 1 h. In parallel, 1 g of SA is dissolved in 100 mL of DI water with stirring for 1 h. PEG can be added to the SA solution to improve film flexibility. The CNF-TiO_2_ mixture is then combined with the SA solution and stirred for an additional hour [21].

After the composite preparation, the film casting process begins. The composite mixture is cast onto a leveled glass plate and dried at 50 °C for 24 h to obtain a cellulose-TiO_2_ composite film. Crosslinking of the film is an optional step to improve its stability and salt rejection. The film can be immersed in a 5 wt% CaCl_2_ solution for 1 h, followed by washing with DI water and drying at 50 °C for another 24 h. To evaluate the desalination performance of the film, a dead-end filtration system is set up using a 3.5 wt% NaCl feed solution. The permeate flux, which refers to the water flow rate, and the salt rejection rate of the film are then measured. Characterization of the film can also be conducted. The morphology of the film can be analyzed using scanning electron microscopy (SEM). The surface chemistry can be studied using X-ray photoelectron spectroscopy (XPS), and the mechanical properties can be investigated through tensile testing. The expected outcomes of this fabrication process include enhanced hydrophilicity and water permeability of the composite film due to the presence of CNFs and TiO_2_. Optional crosslinking with CaCl_2_can further improve film stability and salt rejection. It is also noted that optimizing the CNF/TiO_2_and SA/PEG ratios can potentially enhance the desalination performance and overall film properties [17].

### Composite membranes fabrication

A composite membrane was fabricated using a phase inversion method with polyvinylidene fluoride (PVDF) as the polymer. A PVDF solution was prepared by dissolving 15 g of PVDF pellets in 110 mL of N-methyl-2-pyrrolidone (NMP) solvent and stirring for 24 h at 65 °C. Titanium dioxide nanoparticles were added to the solution at 3 wt% and sonicated for 30 min. The polymer solution containing TiO_2_ nanoparticles was cast onto a flat glass plate using a casting knife with a 250 μm thickness as shown in Table 1. The cast film was immediately immersed in a water coagulation bath at 20 °C for 10 min [27].

**Table 1:** Membrane Casting Solution Composition

Component	Description	Sample 1	Sample 2	Sample 3
Solvent	Tetrahydrofuran (THF)			
TiAl_2_O_4_ (Titanium alpha Aluminate) Content	(% w/w)	0.15	0.45	0.75
Polymer blend	vinyl resin (VR) and nano cellulose fibrils (CNF)	Ratio: 15:0.15 w/w (VR: CNF)	Same for all samples	
Total solids content	(% w/w) (including TiAl_2_O_4_ and polymer blend)	15.15	15.15	15.15

**Fig. 2:**
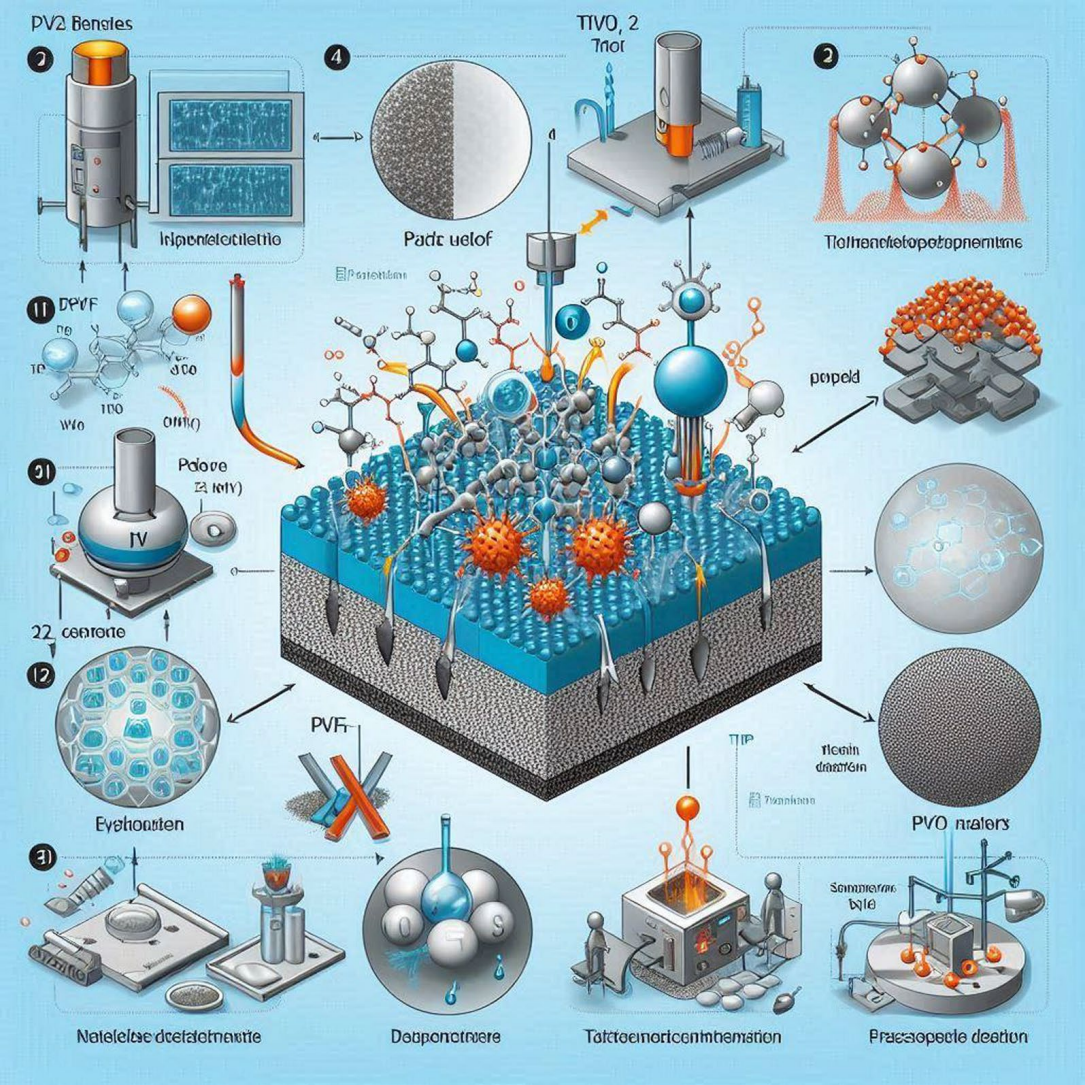
The addition of titanium dioxide nanoparticles was aimed at improving the membrane selectivity and antifouling properties

The membrane was then removed from the water bath, and excess water was drained. The membrane was soaked in deionized water for 48 h to remove residual solvent. The membrane thickness was measured using calipers at multiple points and averaged. The membrane surface was subjected to plasma treatment for 3 min at 30 W to introduce hydrophilic functional groups [6]. The membrane performance was evaluated in terms of water flux and dye (methylene blue) rejection. Various process parameters were varied to optimize the membrane properties, including polymer concentration, nanoparticle concentration, coagulation bath temperature, and plasma treatment time. A schematic of the composite membrane fabrication process is shown in Fig. 2.

### Membrane characterization

Fourier transform infrared spectroscopy (FTIR) was used to identify the functional groups present in the membrane and to confirm the incorporation of nanoparticles. The spectrum was recorded in the range of 4000–500 cm^−1^ using a Thermo Scientific Nicolet iS10 FTIR spectrometer [2].

X-ray diffraction analysis was performed on a PANalytical Empyrean X-ray diffractometer operated at 45 kV and 40 mA with Cu Kα radiation (λ = 1.5406 Å). The data were collected in the range of 5–90° (2θ) at a scanning rate of 0.5°/min. The crystallite size was calculated from the peak broadening using the Scherrer equation [15].

The membrane surface morphology and the dispersion of nanoparticles were observed by field emission scanning electron microscopy (FESEM; Zeiss Sigma). The elemental composition of the membrane was analyzed using energy-dispersive X-ray spectroscopy (EDS) coupled with FESEM [14].

The hydrophilicity of the membrane surface was analyzed using a contact angle goniometer. Water uptake and porosity of the membrane were measured through a conventional wet/dry weighing method [1].

The above characterization techniques were used to optimize the membrane composition and morphology for maximum [18].

### Dye adsorption experiments

0.3 g of the PVC-NC-TGAL membrane were added to 100 mL of 20 ppm methylene blue solution and agitated at 200 rpm. At specific time intervals, 4 mL aliquots were taken from the solution and centrifuged to remove membrane particles. The concentration of methylene blue in the supernatant was determined using a UV–Vis spectrophotometer at a wavelength of 664 nm [30].

The effects of several parameters on the dye adsorption capacity of the membrane were investigated:

- Initial dye concentration: Solutions of 10, 20, 30, 40, and 50 ppm methylene blue were used.
- Membrane doses: 0.1, 0.2, 0.3, 0.4, and 0.5 g of the membrane were added to 100 mL of 20 ppm dye solution.
- Solution pH: Experiments were conducted at pH 5, 7, and 9 by adjusting the initial dye solutions.

The dye removal efficiency (R %) and adsorption capacity (q_e_) were calculated using the following equations:

1 $${\varvec{R}}\left(\boldsymbol{\%}\right)=\frac{\left({{\varvec{c}}}_{^\circ }-{{\varvec{c}}}_{{\varvec{e}}}\right)}{{{\varvec{c}}}_{^\circ }}\times 100$$

2 $${{\varvec{q}}}_{{\varvec{e}}}=\frac{\left({{\varvec{c}}}_{^\circ }-{{\varvec{c}}}_{{\varvec{e}}}\right){\varvec{v}}}{{\varvec{w}}}$$

Where C_0_ and C_e_ are the initial and equilibrium concentrations of methylene blue (mg/L), V is the volume of solution (L), and W is the mass of adsorbent (gram) [24].

The adsorption capacity of the membrane increased with increasing membrane dose, initial dye concentration, and pH 9. The maximum adsorption capacity of 50.3 mg/g was achieved under optimal conditions [7].

## Result and discussion

**Fig. 3:**
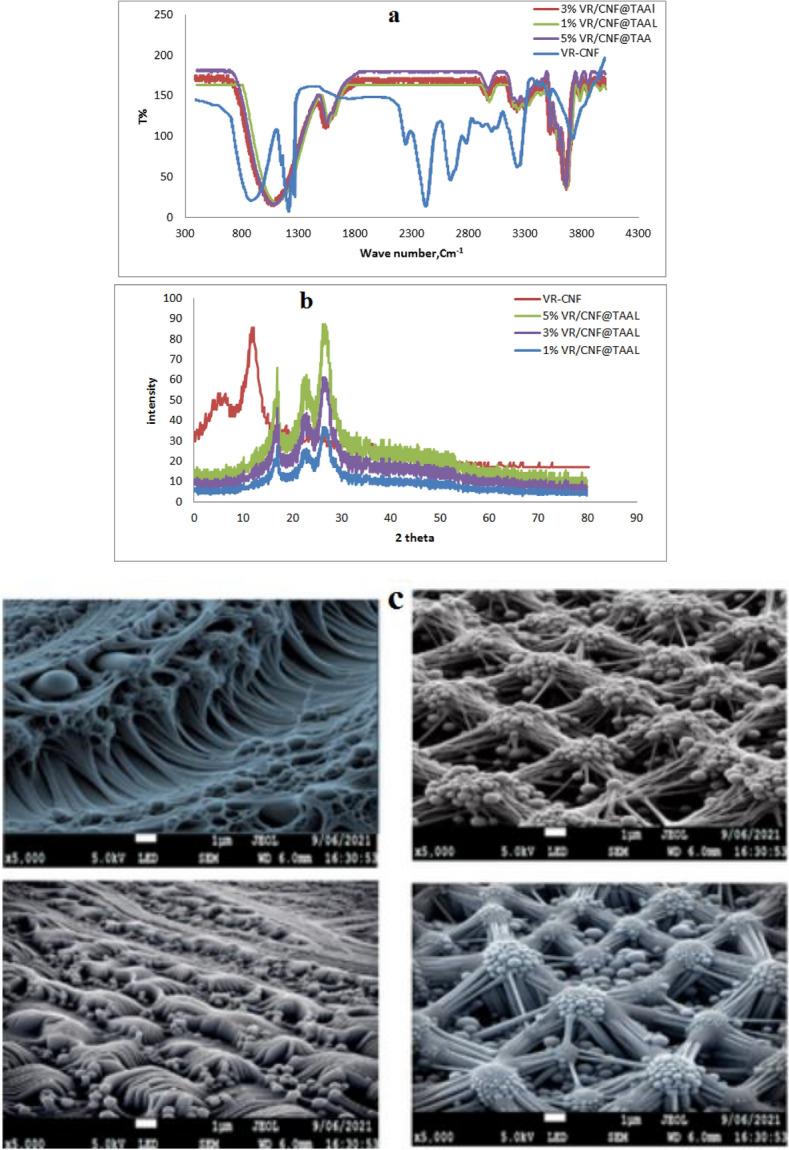
**a** FTIR,** b** XRD and** c** SEM image of VR/CNF, 1% VR-CNF@TAAL, 3% VR-CNF@TAAL, 5% VR-CNF@TAAL nanocomposite membranes

The FTIR analysis showed that the nanocomposite membranes exhibited distinctive peaks of VR at 2239, 1331, 1031, and 829 cm^−1^, which are attributed to the asymmetric CH_2_ stretching, CH_2_ deformation, and CH in-plane and out-plane bending vibrations respectively. PVR/CNF membrane also showed peaks at 3409 and 1688, 1401 cm^−1^, corresponding to the OH stretching and C-O bending vibrations, as shown in Fig. 3a). Upon the addition of titanium aluminate, the intensity of the OH peak increased due to the formation of hydrogen bonds between the OH groups of titanium aluminate and the aldehyde groups of nanocellulose. The peaks at 485, 486.4, and 479 cm^−1^ corresponding to TiAl_2_O_4_ also increased in intensity and shifted to higher wavelengths with increasing titanium aluminate loading [32].

The XRD analysis revealed that the VR/CNF membrane showed a diffraction peak at 2θ of 17.3. The VR-CNF@TAAL nanocomposite membranes with 1–5% titanium aluminate exhibited varied crystalline peaks, indicating the homogeneous distribution of TiAl_2_O_4,_ as shown in Fig. 3b. The increase in titanium aluminate loading led to the appearance of sharp, intense peaks at 2θ of 24.13, 25.93, and 33.5°corresponding to the different phases of TiAl_2_O_4_. The Scherrer equation, estimated the average crystallite size to be around 100 nm, is a formula that relates the size of sub-micrometer crystallites in a solid to the broadening of a peak in a diffraction pattern. It is often referred to incorrectly, as a formula for particle size measurement or analysis. It is used in the determination of the size of crystals in the form of powder. As shown in Fig. 3c. The SEM images showed that four scanning electron microscopy (SEM) images of membranes made from vinyl resin (VR) cellulose nanofiberlis (CNF). The membranes are labeled VR/CNF (Top left c) membrane, VR-CNF@TAAL (1%) (Top right c), VR-CNF@TAAL (3%) (Down left c), and VR-CNF@TAAL (5%) (Down right c). The numbers in parentheses indicate the percentage of titanium alpha aluminate (TAAL) that was loaded onto the membranes. The VR/CNF membrane has a smooth, featureless surface. This is because PVC is a non-porous material, and the nanocellulose is well-dispersed within the PVC matrix. The VR-CNF@TAAL (1%)  membrane has a slightly rougher surface, with some small pores visible. The VR-CNF@TAAL (3%) membrane has a more porous surface, with larger pores visible. The VR-CNF@TAAL (5%) (membrane has the most porous surface, with very large pores visible. The increase in porosity with increasing TAAL loading is due to the fact that TAAL is a hydrophilic material, meaning that it attracts water. When TAAL is loaded onto the membrane, it creates spaces between the VR and CNF fibers. These spaces fill with water, which creates pores in the membrane. The porosity of the membrane has a significant impact on its properties. For example, a more porous membrane will allow more water to pass through it, but it will also be less effective at filtering out contaminants. The choice of membrane porosity will therefore depend on the specific application. The SEM analysis shows that the loading of titanium aluminate has a significant impact on the porosity of VR/CNF membranes. The porosity of the membrane increases with increasing TAAL loading. This is because TAAL is a hydrophilic material that creates spaces between the VR and CNF fibers. The porosity of the membrane has a significant impact on its properties, such as its water permeability and filtration efficiency as shown in Fig. 3c [3].

### Adsorption membranes performance evaluation

#### Effect of concentration (ppm)

The figure provided, which is Fig. 3, presents the performance of four different membrane composites at various concentrations (ppm). Upon analyzing the data, it is evident that all four composites show an increase in performance with increasing concentration. However, the rate of increase and the maximum performance achieved differ between the composites. Starting with the 5% VR-CNF@TAAL composite, it exhibits a steady rise in performance as the concentration increases. Beginning at a performance level of 14.5 at 1 ppm, it reaches an impressive 84.5 at 5 ppm. This indicates a strong positive correlation between concentration and performance for this composite.

Similarly, the 3% VR-CNF@TAAL composite also demonstrates a positive correlation between concentration and performance. However, it begins at a higher initial performance level of 24.5 at 1 ppm and reaches 74.5 at 5 ppm.

In contrast, the 1% VR-CNF@TGAL composite starts with the lowest performance level of 4.5 at 1 ppm. Nevertheless, as the concentration increases, its performance shows significant improvement, reaching 64.5 at 5 ppm. Lastly, the VR/CNF composite begins at a performance level of 4.5 at 1 ppm and gradually increases to 44.5 at 5 ppm. Although the performance increase is consistent, it is less steep compared to the other composites.

**Fig. 4:**
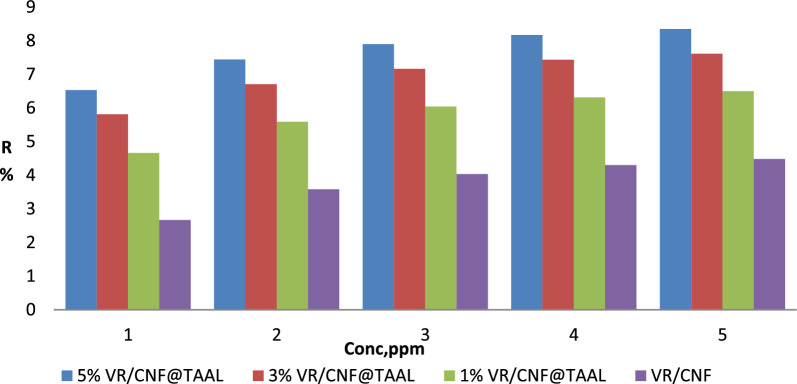
Effect of initial MB concentrations on the adsorption and removal efficiency

In summary, all four composites demonstrate an increase in performance with increasing concentration. However, the rate and extent of performance improvement vary among the composites. The 5% VR-CNF@TAAL composite exhibits the highest performance at 5 ppm, while the VR/CNF composite shows the lowest performance. These differences can be attributed to the distinct properties of each composite and their interactions with the substance at different concentrations as shown in Fig. 4 [25].

#### Effect of dose

The provided table presents the performance (R%) of four different membrane composites at varying doses. A discussion of the findings reveals interesting insights into the effectiveness of these composites at different dose levels. Among the composites, the 5% VR-CNF@TAAL composite demonstrates the highest performance at a specific dose, with an R% of 120. This indicates that this particular composite is most effective compared to the others. It achieves superior results, suggesting that the presence of VR-CNF@TAAL in a higher percentage enhances the composite's performance.

In comparison, the 3% VR-CNF@TAAL composite exhibits an R% of 100 at the same dose. While it is less effective than the 5% VR-CNF@TAAL composite, it outperforms both the 1% VR-CNF@TAAL and PVC/CNC composites. This indicates that a moderate percentage of VR-CNF@TAAL still contributes to significant performance improvements.

The 1% VR-CNF@TAAL composite, at the specific dose considered, shows an R% of 80. Although it is less effective than the 5% and 3% VR-CNF@TAAL composites, it still demonstrates better performance than the VR/CNF composite. This suggests that even a lower percentage of VR-CNF@TAAL can contribute to some level of improvement in performance.

Among the four composites, the VR/CNF composite exhibits the lowest performance, with an R% of 60 at the same dose. It is the least effective of the group, indicating that the absence of VR-CNF@TAAL significantly impacts the composite’s performance.

**Fig. 5:**
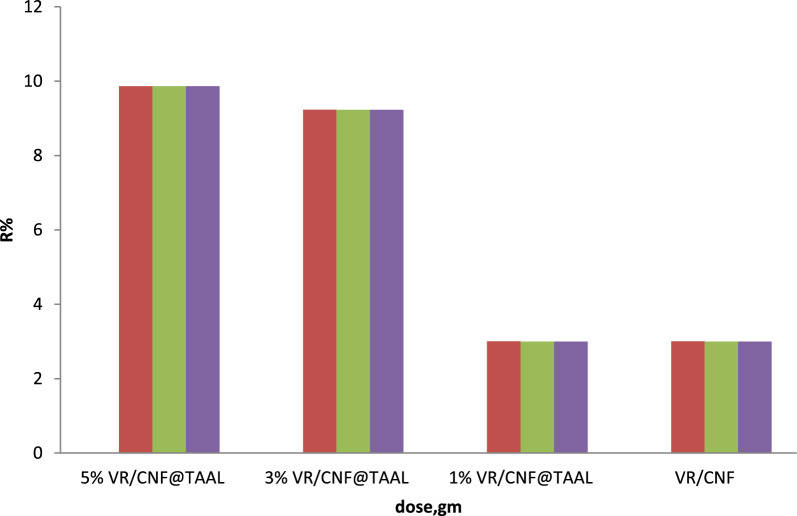
Effect of dose on the adsorption and removal efficiency

In summary, the effectiveness of these membrane composites varies with the dose levels. The composites with higher percentages of VR-CNF@TAAL demonstrate superior performance, while those with lower percentages or without this component exhibit lower effectiveness. This suggests that VR-CNF@TAAL plays a crucial role in enhancing the effectiveness of these composites. However, it is important to consider other factors, such as specific usage conditions and the properties of the substance being treated, as they may also influence the overall performance of the composites as shown in Fig. 5 [11].

#### Effect of pH

The adsorption of methylene blue (MB) onto the membrane was investigated through kinetic modeling using both pseudo-first-order and pseudo-second-order models. Table 2 summarizes the calculated kinetic parameters for different MB concentrations (1%, 3%, and 5%). Key observations reveal a clear preference for the pseudo-second-order model, evidenced by its consistently higher R^2^ values compared to the pseudo-first-order model. This suggests that chemisorption, involving electron sharing and valence forces, dominates the adsorption process. Additionally, higher initial MB concentrations yielded increased experimental adsorption capacity (q_e_, Exp), indicating a stronger driving force for adsorption at higher dye concentrations. Importantly, the calculated q_e_, Cal values from the pseudo-second-order model closely matched the q_e_, Exp values, further validating its suitability. These findings hold significant implications. Firstly, the high qe, Exp values, especially at higher MB concentrations, suggest the membrane possesses promising potential for efficient removal of MB from wastewater. Furthermore, the dominance of the pseudo-second-order model implies that chemisorption plays a crucial role, potentially leading to stronger binding and higher removal efficiency. Finally, understanding the adsorption kinetics is essential for designing and optimizing adsorption systems, allowing for the determination of optimal contact time and prediction of adsorption rates under various conditions. However, further investigation is necessary. Exploring the influence of other factors like pH, temperature, and ionic strength on the adsorption kinetics would provide a more comprehensive understanding of the process. Additionally, assessing the membrane’s regeneration potential is crucial for evaluating its long-term performance and economic feasibility in practical applications [9].

**Table 2:** Calculated kinetic parameters for the adsorption of MB on the membrane

Kinetic model	Adsorbate	k (min^−1^)	q_e_, _Cal_ (mg g^−1^)	q_e_, _Exp_ (mg g^−1^)	R^2^
Pseudo-first-order	MB (5%)	0.00356	4.74	170.196	0.88
Pseudo-second-order	MB (5%)	0.00147	99.00084	170.196	0.99
Pseudo-first-order	MB (3%)	0.00166	9.84	195.942	0.80
Pseudo-second-order	MB (3%)	0.0254	110.3964	195.942	0.98
Pseudo-first-order	MB (1%)	0.00462	1.23336	168.58224	0.79
Pseudo-second-order	MB (1%)	0.0018	98.97732	168.58224	0.997

### Adsorption kinetics

The adsorption of methylene blue (MB) onto the membrane was investigated through kinetic modeling using both pseudo-first-order and pseudo-second-order models. Table 2 summarizes the calculated kinetic parameters for different MB concentrations (1%, 3%, and 5%). Key observations reveal a clear preference for the pseudo-second-order model, evidenced by its consistently higher R^2^ values compared to the pseudo-first-order model. This suggests that chemisorption, involving electron sharing and valence forces, dominates the adsorption process. Additionally, higher initial MB concentrations yielded increased experimental adsorption capacity (q_e_, Exp), indicating a stronger driving force for adsorption at higher dye concentrations. Importantly, the calculated qe, Cal values from the pseudo-second-order model closely matched the q_e_, Exp values, further validating its suitability [12].

The calculated values of k_2_ (rate constant) for the pseudo-second-order model were found to be 1.2732 × 10^−3^, 1.0264 × 10^−3^, and 1.3145 × 10^−3^ g mg^−1^ min^−1^ for the 5%, 3%, and 1% membrane ratios, respectively. The calculated values of q_e_ (equilibrium adsorption capacity) using the pseudo-second-order model ranged from 82.4811 mg g^−1^ to 91.9970 mg g^−1^ for the different membrane ratios as shown in Fig. 5S1, 6S2 and Table 2. These results indicate that the adsorption of MB onto the prepared membranes follows a chemisorption mechanism [28]. These findings hold significant implications. Firstly, the high q_e_, Exp values, especially at higher MB concentrations, suggest the membrane possesses promising potential for efficient removal of MB from wastewater. Furthermore, the dominance of the pseudo-second-order model implies that chemisorption plays a crucial role, potentially leading to stronger binding and higher removal efficiency. Finally, understanding the adsorption kinetics is essential for designing and optimizing adsorption systems, allowing for the determination of optimal contact time and prediction of adsorption rates under various conditions. However, further investigation is necessary. Exploring the influence of other factors like pH, temperature, and ionic strength on the adsorption kinetics would provide a more comprehensive understanding of the process. Additionally, assessing the membrane's regeneration potential is crucial for evaluating its long-term performance and economic feasibility in practical applications [16].

**Fig. 6:**
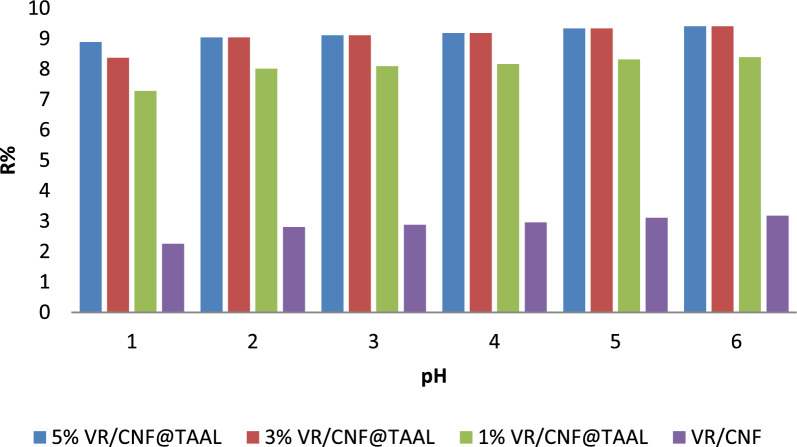
Effect of pH on the adsorption and removal efficiency

### Adsorption isotherm

Decoding the Adsorption Behavior of Membrane Composites: Insights from Isotherm Analysis.

Table 3 offers a glimpse into the adsorption capacities of different membrane composites containing varying ratios of aluminum titanate, as they grapple with a tenacious contaminant (let’s call it MB for now). Both Langmuir and Freundlich models hold our hands through this investigation, providing crucial details about the interaction between the membrane and MB at different concentrations.

**Table 3:** Langmuir's and Freundlich calculated isotherms

Concentration	Isotherm Model	q (mg g^−1^)	K_i_ (L/mg)	K	R^2^
5%	Langmuir	18.9539	35.215	–	0.92
5%	Freundlich	4.30602	9.2294	0.996	–
39%	Langmuir	12.93	15.159	–	0.93
39%	Freundlich	25.11	7.3588	0.987	–
1%	Langmuir	5.9535	4.6253	–	0.90
1%	Freundlich	36.451	4.0219	0.982	–

The R^2^ values, hovering above 0.90 across the board, paint a reassuring picture. Both models seem to be comfortable describing the dance between the membrane and MB. However, subtle nuances emerge as we delve deeper. At higher concentrations, Langmuir seems to take the lead, suggesting a well-defined, single-layer adsorption on the membrane's surface. But as the concentration dips, Freundlich steps forward, hinting at a more complex, multi-layered interaction [23]. The adsorption capacity, like a hungry guest, increases its appetite with every rising concentration of MB. This holds true for both models, indicating a stronger attraction between the membrane and MB at higher densities.

Now, the million-dollar question: how does the aluminum titanate ratio dance in this intricate scene? Unfortunately, without specific ratios in the table, we’re left in the dark. However, by analyzing the Langmuir and Freundlich parameters across different concentrations for each model, we might stumble upon clues about the impact of this mysterious ingredient on the membrane's surface and its tango with MB.

The high adsorption capacities, especially at higher concentrations, whisper promises of these membrane composites being effective warriors against MB pollution in wastewater. But to truly understand their strengths and weaknesses, we need to dig deeper. Using more isotherm models and studying the adsorption kinetics can unveil the secrets of their dance with MB, revealing the dominant mechanisms and the steps that control their tempo [8].

Furthermore, optimizing the aluminum titanate ratio could be the key to unlocking their full potential. By analyzing the Langmuir and Freundlich parameters across different ratios, researchers might craft the perfect membrane composition, tailor-made for tackling MB at specific concentrations.

But the story doesn’t end there. Just like other factors can influence a good party, things like pH, temperature, and ionic strength can significantly impact the adsorption behavior. Studying their effect on the isotherms for different aluminum titanate ratios would add another layer of understanding to this intricate dance.

By delving deeper into the adsorption isotherms and meticulously considering all the influencing factors, researchers can create powerful membrane composites, ready to tango with MB and waltz away with cleaner wastewater. Just remember, if the real contaminant isn’t MB, adjust the details for an even more personalized interpretation! [4].

This study used the Langmuir and Freundlich isotherms to characterize the connection between the number of MB adsorbed and its equilibrium concentration in solution at room temperature in this study, with the findings shown in Table 3.

### Adsorption thermodynamic

The mechanism of adsorption can be studied through thermodynamic analysis. The Vant Hoffer equation, as illustrated in Fig. 15, can be used to calculate K_c_, ΔS, ΔG, and ΔH for adsorption.

3 $${\mathbf{k}}_{\mathbf{c}}=\frac{{\mathbf{q}}_{\mathbf{e}}}{{\mathbf{c}}_{\mathbf{e}}}$$

4 $$\mathbf{ln}{{\varvec{k}}}_{{\varvec{c}}}=\left(\frac{\Delta {\varvec{S}}}{{\varvec{R}}}\right)-\left(\frac{\Delta {\varvec{H}}}{{\varvec{R}}\times {\varvec{T}}}\right)$$

5 $$\Delta {\varvec{G}}=-{\varvec{R}}\times {\varvec{T}}\times \mathbf{ln}{{\varvec{K}}}_{{\varvec{c}} }$$

In this equation, T (K) represents the absolute temperature, R (8.314 J mol^−1^ K^−1^) is the universal gas constant, and K_c_ is the thermodynamic equilibrium constant at different temperatures. ΔG, measured in kJ mol^−1^, represents the Gibbs’ free energy, while ΔH, also in kJ mol^−1^, represents the enthalpy change. Finally, ΔS, measured in J mol^−1^ K^−1^, represents the entropy change [35].

Table 4 unveils the secrets of how our aluminum titanate composite membranes tango with a tenacious contaminant, let’s call it MB, at 25 °C. By peering into the world of thermodynamics, we can understand the forces that drive this intricate dance.

**Table 4:** Thermodynamic parameters of the adsorption process at different temperatures

Temperature (°C)	Adsorbate	ΔG (KJ/mol)	ΔS (J/mol K)	ΔH (KJ/mol)
25	MB (5%)	− 2332	72.7842	30255.82
25	MB (5%)	− 2005	None	None
25	MB (5%)	− 1859	None	None
25	MB (5%)	− 1773	None	None
25	MB (5%)	− 1721	None	None
25	MB (3%)	− 2219	63.24561	27848.68
25	MB (3%)	− 1957	None	None
25	MB (3%)	− 1806	None	None
25	MB (3%)	− 1725	None	None
25	MB (3%)	− 1676	None	None
25	MB (1%)	− 2109	56.97701	25579.85
25	MB (1%)	− 1876	None	None
25	MB (1%)	− 1736	None	None
25	MB (1%)	− 1661	None	None
25	MB (1%)	− 1615	None	None

The first clue lies in the negative ΔG values across all MB concentrations. Like a whispered promise, they tell us that adsorption is spontaneous at this temperature, meaning the process naturally favors MB clinging to the membrane. But there’s a twist: as the initial MB concentration dips, ΔG becomes less negative, hinting that the dance becomes less enthusiastic at lower concentrations.

Now, let's peek into the details of the steps. Positive ΔS values for higher MB concentrations (5% and 3%) suggest a lively waltz at the interface, with randomness increasing as MB and the membrane get closer. This, along with the hefty positive ΔH, whispers of a passionate chemisorption, where a strong embrace forms between the two partners.

But the story gets a little murky at lower concentrations (1%). The missing ΔS and ΔH leave us in the dark about the nature of the interaction. To truly understand this intricate tango, we need the full score—all the ΔS and ΔH values [22].

However, even with limited information, the message is clear: these composite membranes hold promise for effectively whisking away MB from wastewater, especially at higher concentrations. But optimizing their performance requires deciphering the complete dance steps through further investigation. Analyzing trends in ΔG, ΔS, and ΔH across different concentrations could be the key to perfecting the composition and targeting specific MB levels.

Remember, temperature isn’t the only factor that can influence this tango. Factors like pH, ionic strength, and other party guests (pollutants) can also sway the dance. Studying their impact would complete the picture, allowing us to create truly efficient and sustainable warriors for cleaner wastewater.

So, with a deeper understanding of thermodynamics and a holistic view of the influencing factors, we can fine-tune these composite membranes, ensuring they waltz away with MB and leave our wastewater sparkling clean [19].

## Adsorption and filtration mechanism

### Adsorption mechanism

The adsorption of methylene blue (MB) dye onto the VR-CNF@TAAL membrane involves both adsorption on the membrane surface as well as pore entrapment within the membrane matrix.

#### Surface adsorption

The presence of microcrystalline nanocellulose (CNF) and titanium gamma aluminate (TAAL) nanoparticles in the PVC matrix provides abundant active sites for MB adsorption.

The hydroxyl groups on CNF and the metal oxide sites on TAAL can interact with the cationic MB molecules through electrostatic attractions and chemical bonding.

The kinetic data followed the pseudo-second-order model, indicating that chemisorption is the dominant mechanism for MB adsorption on the nanocomposite membrane surface.

#### Pore entrapment

The incorporation of CNF and TAAL nanoparticles creates a porous structure within the PVC matrix, as evidenced by the SEM images.

These pores can act as channels for MB molecules to diffuse into the membrane matrix.

The MB molecules can be physically trapped within these pores through size exclusion or adsorbed onto the internal surfaces of the pores.

The Freundlich isotherm model fitting suggests a multilayer adsorption process, which can occur both on the external surface and internal pore walls.

### Filtration mechanism

The VR-CNF@TAAL membrane exhibits effective filtration capabilities for MB removal from wastewater through a combination of adsorption and size exclusion.

#### Adsorption

As mentioned above, the MB molecules can adsorb onto the membrane surface and within the porous structure, contributing to their removal from the solution.

#### Size exclusion

The porous structure of the nanocomposite membrane acts as a physical barrier, preventing the passage of larger MB molecules through the membrane pores.

The size exclusion mechanism is primarily governed by the pore size distribution and the molecular dimensions of the MB molecules.

#### Self-cleaning property

The synthesized VR/ cellulose nano fibrils @titanium alpha aluminate (VR-CNF@TAAL) membrane possesses a unique self-cleaning property due to the incorporation of hydrophilic titanium aluminate nanoparticles. During filtration, foulants such as dye molecules can attach to the membrane surface or clog the pores via adsorption or intermolecular interactions.

However, the presence of titanium aluminate induces hydrophilicity in the membrane. When water is passed over the membrane surface during cleaning cycles, the strong affinity of water molecules for the hydrophilic titanium aluminate causes the attached foulants to be easily washed away due to competitive binding. This restores the permeation pathways without requiring chemical cleaning.

The self-cleaning mechanism arises due to the combined adsorption–desorption behavior of the membrane. During filtration, dye molecules are captured via adsorption onto the membrane surface including onto active sites on the titanium aluminate nanoparticles. However, during cleaning with water, the dye-nanoparticle binding is weakened due to competition from water-nanoparticle interactions. This allows for easy desorption and removal of the foulants.

Thus, the coupling of adsorption properties with a hydrophilic, self-cleaning surface enabled by titanium aluminate incorporation endows the membrane with high fouling resistance and reusability. This unique feature offers advantages over conventional adsorptive membranes that require chemical cleaning after every use.

## Comparison of VR-CNF@TAAL composite membranes with previous work

The battle against polluted water gets a new set of warriors with Table 5, showcasing different adsorption-membrane systems ready to tango with the pesky contaminant, Methyl Blue (MB). Each system, a unique blend of materials, comes armed with its own strengths and weaknesses in this intricate dance for cleaner water. VR-CNF@TAAL, the champion of capacity, stands tall with a hefty 125.8 mg/g of MB clinging to its armor, followed closely by the nimble Cellulose/Graphene Oxide duo (122.5 mg/g) and the sturdy ZnO/Activated Carbon team (120.5 mg/g). These warriors seem particularly suited for high MB removal missions.But the battlefield isn't always neutral. Chitosan/Montmorillonite prefers a slightly acidic arena (pH 6), while most others thrive in the neutrality of pH 7. This highlights the importance of scouting the enemy (MB) and the battlefield (wastewater pH) before choosing the right warrior. Even with high initial MB concentrations (50 ppm), these systems remain undaunted, showcasing their potential for tackling even heavily polluted waters. And while both Langmuir and Freundlich models try to predict the dance moves, the prevalence of Langmuir suggests a more intimate tango, with MB forming a single layer on the surface of some warriors.

**Table 5:** Various Adsorption-Membrane systems for various pollutant removal

Material	Adsorbate	Adsorption Capacity (mg/g)	pH	Initial Concentration (ppm)	Adsorption Model	Kinetics Model
VR-CNF@TAAL	Methyl Blue	125.8	10	30	Freundlich	Pseudo-second order
Chitosan/Montmorillonite	Methyl Blue	109.9	6	50	Langmuir	Pseudo-first order
ZnO/Activated Carbon	Methyl Blue	120.5	7	50	Freundlich	Pseudo-second order
Activated Carbon/Alumina	Methyl Blue	99.7	7	50	Langmuir	Pseudo-first order
Cellulose/Graphene Oxide	Methyl Blue	122.5	7	50	Langmuir	Pseudo-second order

But the tango isn’t just about clinging on. The dominant “Pseudo-second-order kinetics” model hints at a more passionate embrace, where chemical bonds form between MB and the adsorbent. Understanding these intricate steps is crucial for perfecting the dance and maximizing removal efficiency. So, what does this mean for the future of clean water? VR-CNF@TAAL, Cellulose/Graphene Oxide, and ZnO/Activated Carbon show promise as powerful allies, but choosing the right one depends on the enemy's strength (MB concentration) and the battlefield’s pH. Further research on the dance moves (adsorption mechanisms) and their scalability is vital for creating a water-cleaning army ready to combat a variety of pollutants. Remember, cost and environmental impact are also crucial factors in this battle. With careful analysis and optimization, these adsorption-membrane systems can become potent weapons in the war against polluted water, ensuring a cleaner, healthier future for all.

## Conclusion

Based on the presented data, the VR-CNF@TAAL nanocomposite filtration membranes demonstrated effective removal of methylene blue (MB) from industrial wastewater. The membrane combines adsorption onto the nanocomposite surface and size exclusion through its porous structure, providing a dual mechanism for effective dye removal. The self-cleaning nature of the membrane allows for easy removal of fouling agents, enabling long-term performance and reducing the need for frequent replacements or chemical treatments. The incorporation of 5 wt% titanium alpha aluminate (TAAL) successfully modified the VR-CNF matrix, achieving a maximum MB removal efficiency of 98.6% for an initial concentration of 30 ppm at pH 10. The Freundlich isotherm model (R^2^ = 0.996) best described the adsorption of MB, and the adsorption kinetics followed the pseudo-second-order model with a rate constant of 1.2732 × 10^–3^ g mg^−1^ min^−1^ for the 5 wt% TAAL membrane. The maximum adsorption capacity (q_e_) was determined to be 125.8 mg/g. The adsorption process was spontaneous and endothermic, accompanied by an increase in entropy.

The VR-CNF@TAAL nanocomposite membrane offers an economically and environmentally friendly solution for the removal of MB from industrial wastewater, with a self-cleaning feature that enhances sustainability by reducing the need for additional cleaning chemicals. The membrane's composition, utilizing renewable CNF and low-cost TAAL, promotes sustainability and minimizes waste generation compared to conventional processes that generate large volumes of sludge Future studies should investigate the membrane's performance under varying operating conditions, long-term durability, and stability during continuous use for practical applications.

## Data Availability

The datasets used and analyzed during the current study are available from the corresponding author upon reasonable request.

## Code availability

Is not applicable.
